# Recurrent Euglycemic Diabetic Ketoacidosis Precipitated by Diabetic Myonecrosis in a Patient with Type 1 Diabetes Mellitus

**DOI:** 10.1016/j.aed.2025.03.002

**Published:** 2025-04-10

**Authors:** Keerthana Haridas, Timothy Iafe, Megan McConnell

**Affiliations:** 1 Division of Endocrinology, Diabetes and Metabolism, University of California Los Angeles David Geffen School of Medicine, Los Angeles, California; 2 Department of Radiological Sciences, University of California Los Angeles, Los Angeles, California

**Keywords:** euglycemic diabetic ketoacidosis, myonecrosis, SGLT2 inhibitor

## Abstract

**Background/Objective:**

Euglycemic diabetic ketoacidosis (eDKA) is a well-recognized complication with sodium linked cotransport of glucose-2 inhibitor (SGLT2i) use. Recurrent eDKA is an infrequently described entity. We describe a patient with recurrent eDKA precipitated by diabetic myonecrosis.

**Case Report:**

A 48-year-old male with Diabetes Mellitus treated with empagliflozin and insulin, presented with left thigh pain and anorexia. Physical examination was notable for BMI 16 kg/m^2^ and left thigh tender induration. Laboratory evaluation revealed pH 7.1, bicarbonate 10 mmol/L, anion gap 32 mmol/L, glucose 168 mg/dl, erythrocyte sedimentation rate 67 mm/hr, Creatine Kinase 31 U/L, glucosuria (4+), ketonuria (4+), HbA1c 11.3%, C-peptide <0.5 ng/ml and glutamic acid decarboxylase antibody titer 64.3 IU/ml. He was diagnosed with eDKA due to SGLT2i use. Empagliflozin was discontinued. MRI of the left thigh revealed diabetic myonecrosis. He was treated with insulin infusion leading to eDKA resolution on hospital day 3. On hospital day 5, bicarbonate was 15 mmol/L, anion gap 18 mmol/L, beta-hydroxybutyrate 49.6 mg/dl, glucose 185 mg/dl, glucosuria (4+) and ketonuria (4+). Recurrent eDKA was diagnosed. Insulin infusion was re-started, causing resolution. The patient was treated with cefazolin and underwent surgical debridement of necrotic muscle.

**Discussion:**

The risk of eDKA with SGLT2i use is increased in patients with T1DM with decreased oral intake, surgery or trauma. Although the half-life of empagliflozin is 12 to 14 hours, persistent euglycemic DKA for 7 to 12 days from the last dose has been reported. Persistent glucosuria and ketonuria in this patient with serum glucose below the renal threshold confirmed recurrent eDKA.

**Conclusion:**

eDKA may recur until 2 weeks from last dose of SGLT2i under certain conditions.

**Highlights**

• The risk of euglycemic diabetic ketoacidosis (eDKA) is increased in the setting of decreased endogenous insulin production, decreased oral intake, surgery, trauma, or infection
• eDKA must be suspected even beyond the predicted duration of circulation of sodium-linked cotransport of glucose-2 inhibitor (SGLT2i) if evidence of anion gap metabolic acidosis, persistent glucosuria, and ketonemia (with blood glucose levels below the renal threshold) with no other causes is present
• Type 1 diabetes mellitus must be ruled out before initiating the use of SGLT2i

**Clinical Relevance**

Although eDKA due to SGLT2 inhibitors is a known entity, little information is available regarding the persistence or recurrence of this metabolic complication beyond pharmacokinetic predictions, despite the widespread use of this class of drugs. This knowledge would allow timely and appropriate diagnoses and treatments.

## Introduction

Euglycemic diabetic ketoacidosis (eDKA) is a well-recognized but underreported adverse effect associated with the use of sodium-linked cotransport of glucose-2 inhibitors (SGLT2i).^1^^,^^2^ It is characterized by euglycemia (blood glucose level <250 mg/dL), metabolic acidosis (arterial pH <7.3 and serum bicarbonate level <18 mEq/L), and ketonemia.^1^, ^2^, ^3^ The incidence ranges from 0.16 to 0.76 per 1000 patient-years in type 2 diabetes mellitus (T2DM) and accounts for a third of all DKA cases.^3^ eDKA occurs due to a combination of multiple factors—glucosuria leading to decreased insulin levels and increased glucagon levels, causing lipolysis and ketogenesis, intravascular volume depletion as well as increased reabsorption of ketone bodies in the proximal convoluted tubule.^2^^,^^4^

eDKA is treated with aggressive intravascular volume repletion, insulin, and discontinuation of SGLT2i.^2^

The objective of this case report was to describe a 48-year-old man with DM treated with empagliflozin who developed recurrent eDKA precipitated by diabetic myonecrosis.

## Case Report

A 48-year-old man with poorly controlled DM with a hemoglobin A1c of 11.3% (15.4 mmol/L) was admitted to the hospital with left thigh pain and anorexia.

He was diagnosed with presumed T2DM at 37 years of age. At the time of diagnosis, the patient was started on several oral hypoglycemic agents. He was started on multiple daily injections of insulin 5 years before presentation and on empagliflozin 4 years before presentation. The patient had no known complications of DM. He was admitted to the hospital for high anion gap metabolic acidosis noted on a laboratory investigation conducted during a visit to his outpatient endocrinologist. Results are tabulated in Table 1 (Table 1, “Day 0”). On presentation to the hospital, his medications included metformin, empagliflozin, pioglitazone, and a basal-bolus insulin regimen. The patient took empagliflozin until the day before hospitalization.

**Table 1:** Timeline of Metabolic Derangements Indicating the Resolution and Recurrence of eDKA

Day of admission	Day 0	Day 3	Day 5	Day 9
Days since the use of SGLT2i	1	2	6	10
Duration of preceding insulin infusion (h)	0	36	0	48
WBC (4.16-9.95 × 10^3^ per microL)	10.76	8.3	13.22	8.69
Venous blood pH (7.3-7.4)	7.1	7.38	7.44	7.47
Venous blood pCO2 (37-65 mm Hg)	40	38	31	45
Serum bicarbonate (20-30 mmol/l)	10	25	15	28
Anion gap (8-12 mmol/L)	32	11	18	9
Lactate (3-18 mg/dL)	17	NA	8	9
Serum glucose (65-99 mg/dL)	168	120	185	201
β-hydroxybutyrate (≤3mg/dL)	NA	NA	49.6	NA
Urine glucose	4+	NA	4+	2+
Urine ketone	4+	NA	4+	2+

Abbreviations: eDKA = euglycemic diabetic ketoacidosis; SGLT2i = sodium-linked cotransport of glucose-2 inhibitor; WBC = white blood cell.

At the time of hospitalization, the patient endorsed polyuria, anorexia, and pain over his left thigh. He denied abdominal pain, nausea, or vomiting. Physical examination was notable for a temperature of 99 °F, blood pressure of 128/70 mm Hg, heart rate of 87 bpm, respiratory rate of 18 bpm, a body mass index of 16 kg/m^2^, and left lateral thigh induration with tenderness. Relevant vital signs during the patient’s hospitalization are summarized in Table 2. The patient’s laboratory test results revealed a white blood cell count of 11.28 × 10^3^ cells per microL, an erythrocyte sedimentation rate of 67 mm/h (≤12 mm/h), GFR of >90 mL/min, a creatine kinase level of 31 U/L (63-473 U/L), a venous pH of 7.1 (7.35-7.45), a bicarbonate level of 10 mmol/l (20-30 mmol/l), a serum glucose level of 168 mg/dL (65-99 mg/dL), an anion gap of 32 (8-12), 4+ urine glucose, and 4+ urine ketones. Serum β-hydroxybutyrate was not measured at the time. He was also found to have C-peptide level of <0.5 ng/mL, glutamic acid decarboxylase antibody level of 64.2 IU/mL (0-5 IU/mL), and islet cell antibody <5.4 U/mL (0-7.4 U/mL). Magnetic resonance imaging of the left thigh revealed a fluid collection measuring 11 cm, favored to reflect diabetic myonecrosis (Fig.). He was diagnosed with T1DM and eDKA in the setting of SGLT2 inhibitor use.

**Table 2:** Vital Signs During the Patient’s Hospitalization

Day of hospitalization	BP (mm Hg)	HR (bpm)	RR (bpm)	Temperature (°F)	Spo2 (%)
0	128/70	87	18	99	100
5	132/88	116	20	103.6	97
13	114/74	83	18	99.2	100

Abbreviation: RR = respiratory rate.

**Fig:**
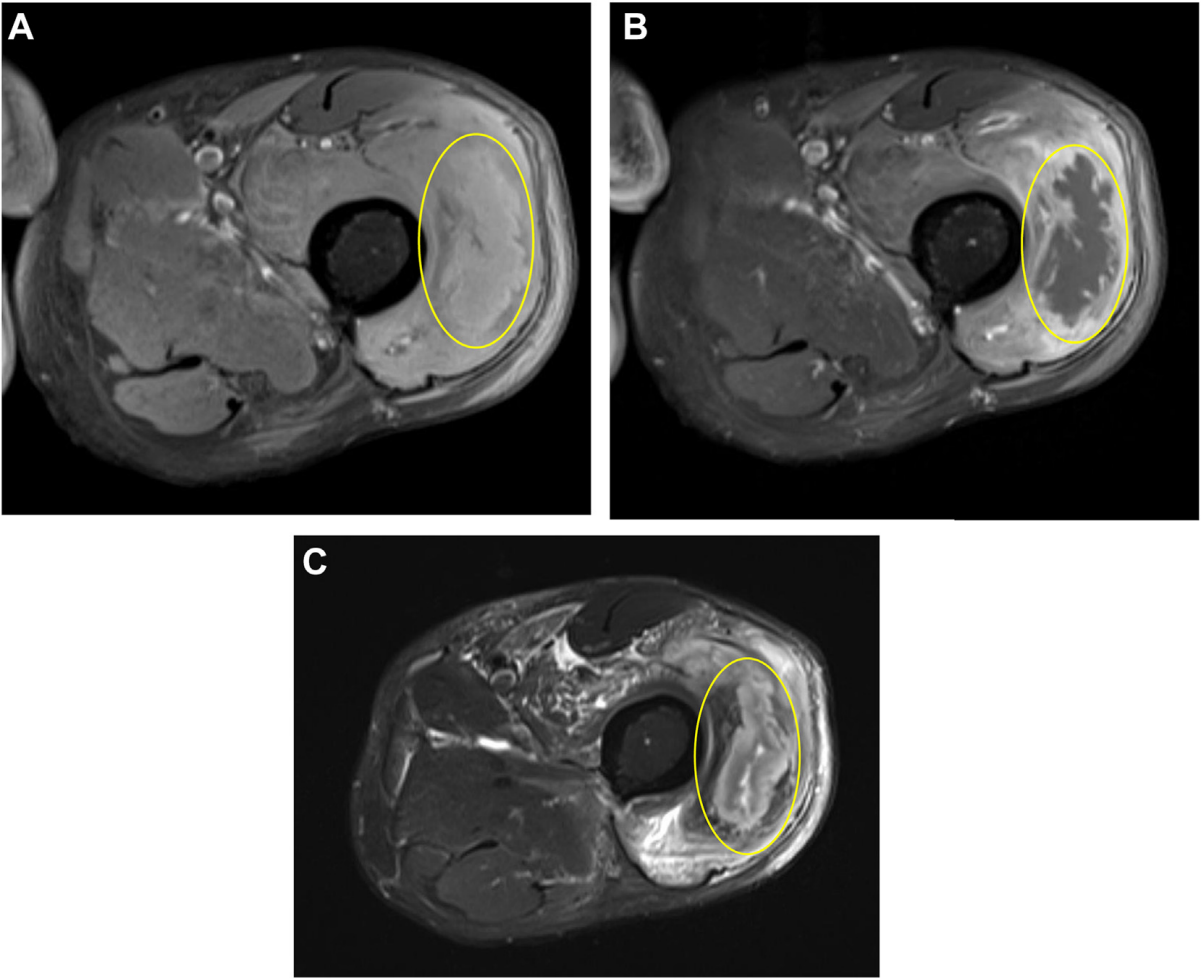
Magnetic resonance imaging of the left thigh depicting diabetic myonecrosis – large complex intramuscular fluid collection within the anterior compartment, which is internally nonenhancing with mildly T1 hyperintense and mixed T2 signal characteristics, consistent with myonecrosis. Also seen is the surrounding nonorganized, multifocal muscular, and soft tissue edema and enhancement, reflecting myositis. *A,* Precontrast T1 fat saturated (FS) image L thigh. *B,* Postcontrast T1 FS image L thigh. *C,* Axial T2 FS image of left thigh. The yellow circle highlights the areas of diabetic myonecrosis.

On hospital day 1, the patient was treated with continuous insulin infusion and intravenous fluids. Analgesics, aspirin, and physical therapy were prescribed for diabetic myonecrosis.

On hospital day 3, the patient had received insulin via infusion for 30 hours. Laboratory test results showed pH of 7.38, bicarbonate level of 25 mmol/l, serum glucose level of 120 mg/dL, and anion gap of 11 (Table 1, “Day 3”). The insulin infusion was discontinued and replaced with a basal-bolus insulin regimen. After transition to subcutaneous insulin, the patient’s serum glucose level ranged between 140 and 180 mg/dL. However, the patient continued to endorse anorexia with associated poor oral intake and worsening thigh pain.

On hospital day 5, the patient developed a fever spike to a *T*_Max_ of 103.6 °F with other vital signs indicated in Table 2 (“Day 5”). Laboratory test results revealed a white blood cell count of 13.2 × 10^3^ cells per microL, pH level of 7.44 with bicarbonate level of 15 mmol/l, anion gap of 18, lactate level of 8 mg/dL (3-18 mg/dL), serum glucose level of 185 mg/dL, and β-hydroxybutyrate 49.6 mg/dL (≤3 mg/dL) (Table 1, “Day 5”). Urinalysis revealed glucosuria and ketonuria (4+ each). Blood cultures grew *Klebsiella pneumoniae* and *Staphylococcus aureus*. Antibiotic treatment with Ceftriaxone was started at this time. Continuous insulin infusion was restarted. On hospital day 9, laboratory test results showed pH level of 7.47, anion gap of 9, bicarbonate level of 28 mmol/l, and serum glucose level of 201 mg/dL. The insulin infusion was discontinued, and the patient was transitioned back to subcutaneous insulin.

On hospital day 13, the patient underwent surgical evaluation of the lateral thigh swelling due to failed conservative management and persistent leukocytosis of 10.55 × 10^3^ per microL. A loculated abscess within necrotic tissue was discovered and was subsequently debrided and drained. The surgical culture grew *S. aureus*. Treatment was completed with cefazolin. The patient was discharged on hospital day 18. Empagliflozin was permanently discontinued, and the patient was started on an insulin pump in the outpatient setting, leading to a decrease in his hemoglobin A1c to 8% (10.2 mmol/L).

## Discussion

SGLT2i act on the SGLT2 channels in the proximal renal tubule and cause renal excretion of glucose.^5^ This glucosuria results in a condition of relative insulin deficiency and glucagon excess, which stimulates lipolysis and the formation of ketone bodies from free fatty acids.^2^^,^^3^ SGLT2i also cause the renal reabsorption of ketone bodies, exacerbating ketonemia.^2^^,^^3^^,^^5^ The likelihood of eDKA is higher in patients with a low body mass index due to low glycogen stores, with low endogenous insulin production, during pregnancy, postbariatric surgery, and with concurrent alcohol use.^2^ It is estimated that between 5% and 12% of patients with T1DM present with eDKA due to the use of SGLT2i.^3^ In our patient, underlying T1DM, persistently decreased oral intake, diabetic myonecrosis, and abscess formation leading to bacteremia precipitated both instances of eDKA.

Although eDKA has been reported in the literature since 2015, there are few case reports of recurrent or persistent eDKA for an interval longer than the duration of circulation of the drug as predicted by pharmacokinetics. The mean half-life of empagliflozin is measured to be between 10.3 and 18.8 hours in multiple-dose studies.^6^ We predict the excretion of the drug from the body in about 72 hours (4-5 ×*t*½), particularly in a patient with intact renal function. However, our patient was noted to have significantly positive glucosuria and ketonuria with serum glucose levels around the renal threshold level (180 mg/dL) about 144 hours after empagliflozin was discontinued. This may be explained by prolonged target binding, ie, extended action of the drug at its binding site.^7^ There is evidence to suggest that the in-vivo duration of drug action not only depends on the pharmacokinetic properties of plasma half-life and the time needed to equilibrate between the plasma and the tissue of action but that it is also influenced by long-lasting target binding and rebinding. Local phenomena can cause the drug to accumulate near the target and/or hinder its movement away from that target, leading to prolonged target binding and downstream action.^8^ These factors can lead to prolonged binding of these drugs to sodium-linked cotransport of glucose channels leading to persistent glucosuria and may predispose to delayed eDKA.

A case report by Pujara et al^9^ reported recurrent eDKA due to dapagliflozin in a patient 9 days after the last dose of dapagliflozin, which is much longer than predicted by the reported half-life of the drug (13 hours). The precipitating cause was not known in this case.^9^ Another case report described persistent glucosuria for 9 days in a patient after discontinuation of canagliflozin despite normal kidney function, demonstrating persistent action of the drug at the targeted channel in the renal tubules. The precipitant, in this case, was low-calorie intake.^10^ A particularly severe instance was reported by Almazrouei et al^11^ describing a patient who needed to undergo hemodialysis due to severe refractory metabolic acidosis, lasting up to 70 hours after initial presentation. A review by Peters et al^12^ demonstrated that 7 of the 13 episodes of eDKA due to canagliflozin were in patients with underlying T1DM and that infection, reduced food intake, increased physical activity, and large recent reductions in insulin doses served as precipitants of eDKA. In our patient’s case, his underlying T1DM, persistently decreased food intake, and diabetic myonecrosis complicated by abscess formation served as triggers for the recurrence of eDKA.

## Conclusion

In conclusion, the risk of recurrent eDKA was increased in our patient due to decreased endogenous insulin production, decreased oral intake, and the presence of diabetic myonecrosis with superimposed infection. Clinicians should consider the diagnosis of T1DM before initiation of treatment with SGLT2i, especially in young patients with poorly controlled DM. A high index of clinical suspicion must be maintained for recurrent eDKA even beyond the predicted duration of circulation of SGLT2i if evidence of anion gap metabolic acidosis, persistent glucosuria, and ketonemia is present.

## Statement of Patient Consent

The patient provided consent for clinical information pertaining to this case to be used in a medical publication.

## Disclosure

The authors have no conflicts of interest to disclose.
